# Impact of Donation After Circulatory Death on Waitlist Duration and Early Outcomes in Heart Transplantation for Patients With Durable Mechanical Circulatory Support

**DOI:** 10.1093/icvts/ivag214

**Published:** 2026-07-30

**Authors:** Takeshi Wada, Joyce Ho, Andrew Belec, Jessica Dominic, Devon Kelly, Shinji Miyamoto, Pavan Atluri

**Affiliations:** Department of Cardiovascular Surgery, Oita University, Idaigaoka 1-1, Hasama-machi, Yufu, Oita 879-5593, Japan; Department of Cardiovascular Surgery, Thomas Jefferson University, 4201 Henry Avenue, Philadelphia, PA 19144, United States; Department of Cardiovascular Surgery, Thomas Jefferson University, 4201 Henry Avenue, Philadelphia, PA 19144, United States; Department of Cardiovascular Surgery, Thomas Jefferson University, 4201 Henry Avenue, Philadelphia, PA 19144, United States; Department of Cardiovascular Surgery, Thomas Jefferson University, 4201 Henry Avenue, Philadelphia, PA 19144, United States; Department of Cardiovascular Surgery, Oita University, Idaigaoka 1-1, Hasama-machi, Yufu, Oita 879-5593, Japan; Department of Cardiovascular Surgery, Thomas Jefferson University, 4201 Henry Avenue, Philadelphia, PA 19144, United States

**Keywords:** heart transplantation, donation after circulatory death (DCD), donation after brain death (DBD), mechanical circulatory support (MCS), waitlist time, United Network for Organ Sharing (UNOS)

## Abstract

**Objective:**

While heart transplantation primarily relies on donors after brain death, the use of donors after circulatory death is increasing to address donor shortages. Although favourable outcomes with donation after circulatory death (DCD) have been reported, its safety in patients bridged with durable mechanical circulatory support (MCS) remains uncertain.

**Design:**

Using the United Network for Organ Sharing database, we identified adult recipients supported with durable MCS who underwent isolated primary heart transplantation between 2019 and 2023. Outcomes were compared between recipients of hearts from donors after brain death and donors after circulatory death.

**Results:**

Among 14 881 heart transplants, 3681 patients were supported with durable MCS, comprising 3374 (91.7%) recipients from donors after brain death and 307 (8.3%) from donors after circulatory death. Compared with recipients of hearts from donors after brain death, those receiving hearts from donors after circulatory death were more often male, had higher body mass index, lower ABO blood group compatibility, fewer pretransplant infections, less extracorporeal membrane oxygenation use, longer ischaemic times (3.5 vs 5.5 hours, *P* < .05), and shorter waitlist times (404.4 vs 336.9, *P* < .05). Survival was similar between groups (90.9% vs 93.2% at 6 months, 89.0% vs 90.2% at 1 year; *P* = .946). Cause of death and other adverse events were also comparable.

**Conclusions:**

Utilizing DCD donors significantly reduces waitlist duration for patients with durable MCS. Although early post-transplant survival was statistically stable, definitive conclusions regarding survival equivalence involve several limitations. Further larger comparative studies with comprehensive donor characteristics are strictly required to evaluate the safety profile of DCD hearts in this high-risk recipient population.

## Graphical abstract

**Figure:**
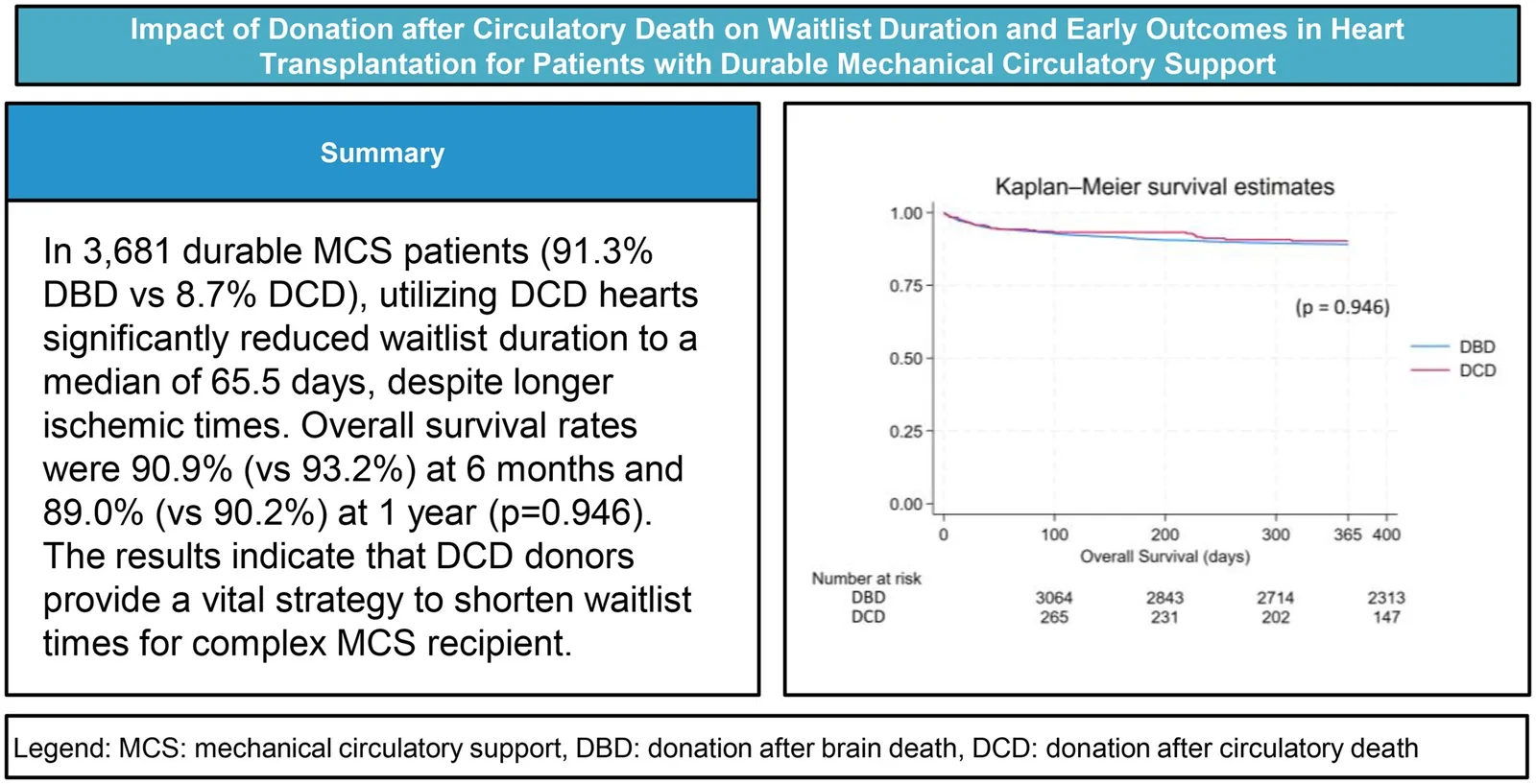

## INTRODUCTION

While heart transplantation has primarily relied on donation after brain death (DBD) donors, the use of donation after circulatory death (DCD) has been rapidly increasing in recent years to address the persistent shortage of available donor hearts.^1^ Among candidates awaiting heart transplantation, patients supported with durable mechanical circulatory support (MCS) have been disproportionately affected by the recent United Network for Organ Sharing (UNOS) allocation policy changes, often receiving lower priority despite improved long-term survival outcomes due to advances in device technology and clinical management that allows greater stability on MCS.

Although DCD heart transplantation has shown excellent outcomes in the general transplant population,^1–3^ its safety and efficacy in recipients supported with durable MCS remains unclear. Conflicting results have been reported,^4–6^ and concerns persist that prolonged support with continuous-flow left ventricular assist devices (CF-LVAD) may lead to diminished post-transplant survival due to device-related adverse events.^7^

In this context, the application of DCD heart transplantation in recipients with durable MCS carries important clinical implications which may improve the outcomes of this complex patient cohort. These patients represent a growing and increasingly complicated subset of the transplant waitlist population. Understanding how DCD allografts perform in this group is critical for optimizing donor-recipient matching and improving outcomes.

To address this knowledge gap, we conducted a retrospective analysis of the UNOS database, establishing waitlist duration as the primary outcome to evaluate the immediate systemic benefit of introducing DCD donors for recipients supported with durable MCS. As secondary outcomes, we examined post-transplant survival and clinical trends. We hypothesized that heart transplantation from donors after circulatory death would yield comparable outcomes to those from donors after brain death in this high-risk population. The integration of such donors significantly augments the donor pool and could provide vital life-saving opportunities, particularly for patients bridged with durable MCS who may otherwise face extended wait times.

## METHODS

UNOS database was used to analyse all DBD and DCD heart transplants between January1, 2019 to December 31, 2023. As the data were fully anonymized, the requirement for individual informed consent was waived. We only included the adult recipients who were provided durable mechanical device at the time of transplant. Continuous variables were analysed using the Mann-Whitney test due to non-normality, which was assessed by the Shapiro-Wilk test. Nominal Variables were analysed using the Chi-square test. The primary end-point of this study was waitlist duration, defined as the time from the patient’s listing for heart transplantation to the date of transplantation. The secondary end-point was post-transplant survival rates at 1 year. Overall survival was estimated using Kaplan–Meier curves, and differences between survival curves were tested using the log-rank test. Inverse probability weighting (IPW) was performed, based on following recipients: age, gender, ABO matching level, body mass index (BMI), prevalence of diabetes, cerebrovascular disease, creatinine level, infection, use of inotrope, intra-aortic balloon pumping (IABP), extracorporeal membrane oxygenation (ECMO) and ventilator. Missing data were assessed for all baseline covariates. The proportion of missingness was minimal, with only 2 missing values for BMI and one for creatinine. These cases were handled using complete case analysis, as the extremely low rate of missing data was unlikely to introduce significant bias. To evaluate the covariate balance between the groups after inverse probability weighting, standardized mean differences (SMDs) were calculated. The SMDs for all baseline covariates are provided in **Table S1**. An SMD of less than 0.1 was considered to indicate an acceptable balance. All analysis was conducted on Stata 17.0. This study utilized de-identified data from the UNOS database. The study protocol was reviewed and approved by the Institutional Review Board (IRB) at Oita University Hospital (Approval Number: 3337).

## RESULTS

**Figure 1.**
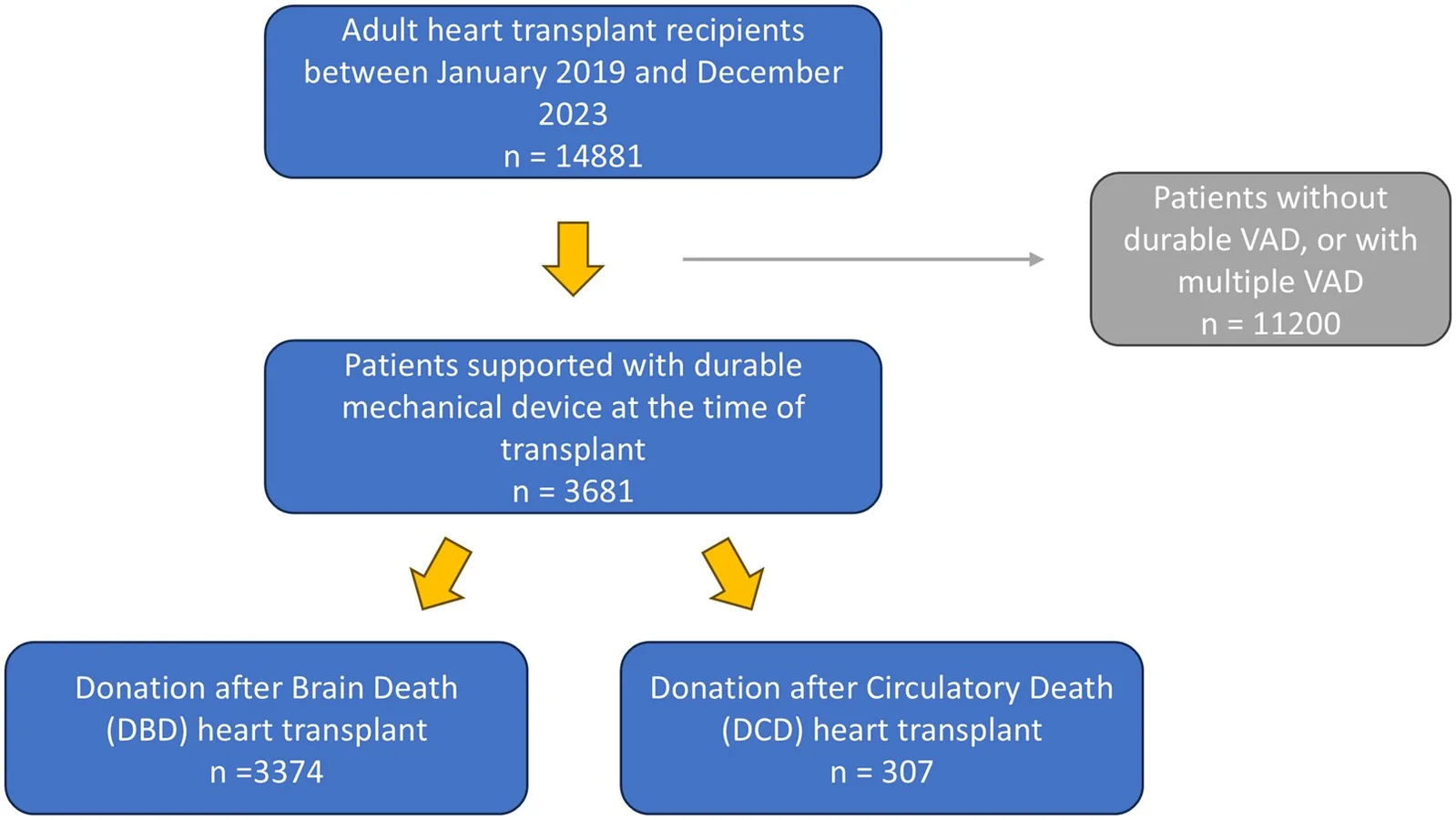
Patients Selection Flow Chart. Patient data was obtained from UNOS database. Durable mechanical devices were defined as HMII, HM3, HVAD, and SynCardia

**Figure 2.**
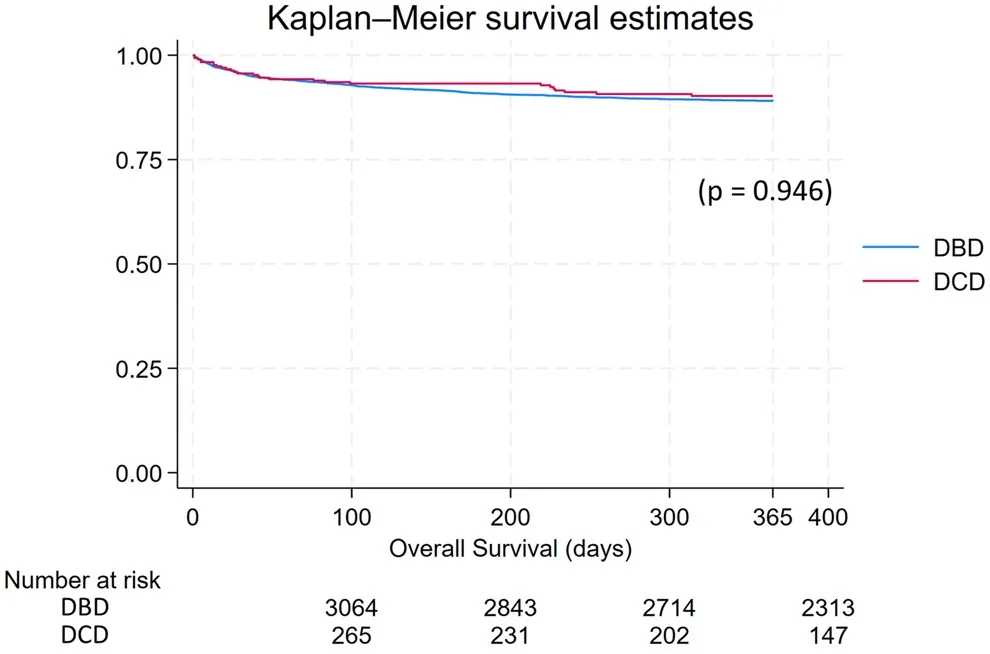
Kaplan–Meier Curve (Original Cohort). Overall survival at 6 months was 90.9% in the DBD group and 93.2% in the DCD group; at 1 year, survival was 89.0% and 90.2%, respectively

A total cohort of 14 881 adult patients underwent orthotropic heart transplant between January 1, 2019 and December 31, 2023 (**Figure 1**). After excluding patients without mechanical support, those with multiple ventricular assist devices (VAD), multiple organ transplants, patients with a previous transplant history—3681 adult heart transplant patients with prior durable mechanical support were identified. This subset was divided into 2 groups, patients who received heart transplants from DBD donors (DBD group) and those who received heart transplants from DCD donors (DCD group). DBD and DCD groups patients comprised 91.7% (*n* = 3374) and 8.3% (*n* = 307), respectively. Regarding donor causes of death, anoxia was the most prevalent in both the DBD and DCD groups (47.4% and 45.6%, respectively), followed by head trauma (36.4% and 44.3%) and cerebrovascular stroke (13.2% and 5.9%). Recipients’ characteristics are shown in **Table 1**. There were significant differences between the 2 groups with regard to gender (22.7% vs 17.3%, *P* = .028), ABO match level (92.2% vs 88.6%, *P* = .025), infection defined by intravenous antibiotics injection (13.7% vs 7.5%, *P* = .006), waitlist days (404.4 vs 336.9, *P* < .01), and heart transplant status level on UNOS criteria (*P* < .01). The types of durable mechanical support were shown in **Table S2**. Post-transplant outcomes are listed in **Table 2**. Ischaemic time (3.48 vs 5.46, *P* < .001) and frequency of transfusions (45.4% vs 51.5%, *P* = .011) demonstrated significant differences, prior to inverse probability weighting. To further evaluate the postoperative clinical stability and safety profile of the DCD cohort, we compared the incidence of major complications and causes of death (**Table 2**). Mortality from liver failure occurred more frequently in DCD group (0.347% vs 4.76%, *P* = .018). Survival following heart transplant in DBD and DCD groups were statistically similar (*P* = .946), at 180 days (90.9% [95% CI, 89.9%-91.9%] vs 93.2% [95% CI, 89.7%-95.6%]), and 1 year (89.0% [95% CI, 87.9%-90.0%] vs 90.2% [95% CI, 86.1%-93.2%]) as shown in **Figure 2**. Donor characteristics and cause of death were shown in **Tables S3** and **S4**, respectively.

**Table 1.** Characteristics

	DBD (*n* = 3374)	Median, (IQR) [Q1, Q3]	DCD (*n* = 307)	Median, (IQR) [Q1, Q3]	*P* value
**Age (years), mean (SD)**	53.3 (SD: 12.2)	56.0 (46.0, 63.0)	53.1 (SD: 12.9)	56.0 (44.0, 64.0)	.972
**Gender (female)**	766 (22.7%)		53 (17.3%)		.028
**ABO match level (Compatible)**	3112 (92.2%)		272 (88.6%)		.025
**BMI (kg/m^2^), mean (SD)**	29.7 (SD: 4.8)	29.7 (26.2, 33.2)	30.2 (SD: 4.8)	30.2 (26.8, 33.7)	.071
**Diabetes**					.608
**No**	2331 (69.1%)		202 (65.8%)		
**Type I**	23 (0.68%)		3 (0.98%)		
**Type II**	981 (29.1%)		100 (32.6%)		
**Cerebrovascular disease**					.052
**No**	3037 (90.0%)		289 (94.1%)		
**Yes**	21 (0.62%)		2 (0.65%)		
**Creatinine, mean (SD)**	1.25 (SD: 0.5)	1.2 (1.0, 1.4)	1.20 (SD: 0.4)	1.1 (0.9, 1.4)	.212
**Dialysis**					.383
**No**	3353 (99.4%)		307 (100%)		
**Haemodialysis**	18 (0.53%)		0		
**Infection**	461 (13.7%)		23 (7.5%)		.006
**Inotropes use**	293 (9.1%)		19 (6.2%)		.133
**Waitlist days (days), mean (SD)**	404.4 (SD: 560.9)	175.5 (36, 547)	336.9 (SD: 523.3)	110 (27, 402)	.002
**IABP use**	29 (0.86%)		1 (0.33%)		.319
**ECMO use**	27 (0.80%)		1 (0.33%)		.839

**Table 2.** Post Transplant Events

	DBD (*n* = 3374)	DCD (*n* = 307)	*P* value
**Overall survival**			.946
**At 30-days**	95.6%	95.6%	
**At 6-month**	90.9%	93.2%	
**At 1-year**	89.0%	90.2%	
**Primary graft failure**	70 (2.1%)	10 (3.3%)	.174
**Acute rejection**	34 (1.0%)	2 (0.7%)	.544
**Chronic rejection**	18 (0.5%)	0	.200
**Ischaemic time (hours), mean (SD)**	3.48 (SD: 1.18)	5.46 (SD: 2.20)	<.01
**Airway dehiscence**	7 (0.2%)	0	.440
**Dialysis**	547 (16.2%)	57 (18.6%)	.476
**Pacemaker implantation**	73 (2.2%)	3 (0.98%)	.225
**Stroke**	196 (5.8%)	15 (4.9%)	.399
**Length of hospital stay, mean (SD)**	27.1 (SD: 33.4)	24.1 (SD: 27.8)	.361
**Cause of death**			
**Cardiovascular related**	80 (13.9%)	6 (14.3%)	.939
**Graft failure related**			
**Infection**	84 (14.6%)	9 (21.4%)	.836
**Cerebrovascular related**	49 (8.5%)	4 (9.5%)	.999
**Haemorrhage related**	15 (2.6%)	3 (7.1%)	.581
**Malignant disease**	33 (5.7%)	1 (2.4%)	.932
**Suicide**	2 (0.4%)	0	.998
**COVID-19 infection**	30 (5.2%)	2 (4.8%)	.999
**Intraoperative death**	1 (0.2%)	0	.999
**Renal failure**	5 (0.87%)	0	.985
**Liver failure**	2 (0.35%)	2 (4.8%)	.018
**Multiple organ failure**	68 (11.8%)	2 (4.8%)	.749

### Inverse probability weighting

**Figure 3.**
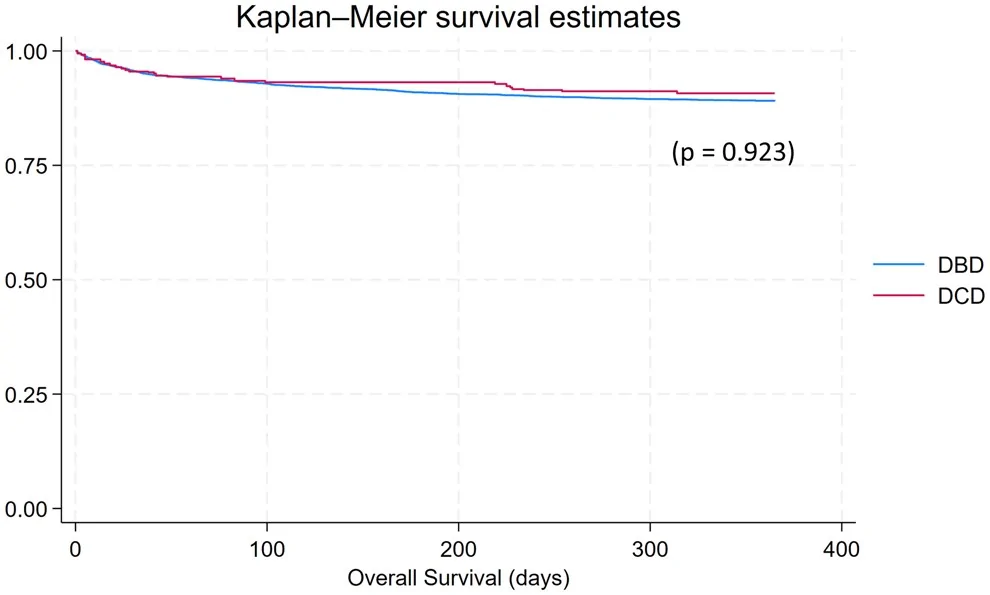
Kaplan–Meier Curve (IPW). Overall survival at 6 months was 88.2% in the DBD group and 93.2% in the DCD group; at 1 year, survival was 85.7% and 90.2%, respectively

Given the limited sample size of the DCD cohort and a post-hoc power of 0.171 to detect subtle survival differences (1.2%), we employed IPW to maximize the statistical rigour of our comparison. Age, Gender, ABO match level, BMI, Diabetes, Cerebrovascular disease, Creatinine level, Infection, Inotropes use, Waitlist days were included in the propensity score calculation. Balances of each covariate are shown on **Table S1**. All standardized differences of each covariate were less than 0.1 after weighted. Overlap plot is shown **Figure S1**. Following weighted Cox regression, the difference between the 2 groups was not statistically significant (HR = 0.98, 95% CI, 0.70-1.39, *P* = .923), as shown in **Figure 3**.

To account for potential selection bias and the confounding effect of donor-level variables, an additional IPW analysis was conducted by including donor age and sex in the propensity score calculation. This was specifically intended to evaluate whether the comparable outcomes were maintained when balancing these key donor characteristics. Weighted Cox regression revealed the 2 groups has no statistically differences (HR = 1.019, 95% CI, 0.71-1.47, *P* = .918).

## DISCUSSION

First, we demonstrated that 1-year post-transplant survival was statistically similar between recipients of DCD and DBD donor hearts (89.0% vs 90.2%, *P* = .946), despite the inherent technical and biological challenges associated with DCD procurement. This finding was further supported by an additional IPW analysis adjusting for donor age and sex, which showed no significant difference between the 2 groups (HR = 1.019; 95% CI, 0.71-1.47; *P* = .918).

Second, the use of DCD donor hearts was associated with a significantly shorter duration on the transplant waitlist compared to the DBD group (404.4 vs 336.9, *P* < .01). This suggests that DCD transplantation provides a critical opportunity for earlier intervention in this high-risk patient population.

Third, while the DCD group experienced a higher incidence of mortality from liver failure (0.347% vs 4.76%, *P* = .018) and required more frequent blood transfusions, these complications did not translate into a significant difference in overall short-to-intermediate survival.

Since the 2018 UNOS policy changes, median wait list times have markedly improved and 1-year waitlist survival has improved from 34.1% to 68.8%.^8^ However, ongoing shortage of donor organs remains a major challenge. In 1967, Barnard and his team performed the world’s first heart transplant using a DCD donor heart. Since this initial experience, heart transplant has sharply shifted to using DBD donors almost exclusively. Nearly 50 years later, the first successful DCD heart transplant in the modern era was reported in Australia in 2015.^9^ Since then, DCD heart transplantation has rapidly expanded worldwide as a strategy to address the organ shortage with improved survival for those waitlisted patients. Madan et al. estimated that DCD donation could increase heart transplants by over 300 annually in the U.S. and over 2000 globally.^10^

Once removed from the waiting list, these patients face a dismal prognosis, with survival rates of only 44% at 30 days and 26% at 1 year. MCS can play a critical role in bridging these patients to transplantation. In addition, heart transplantation and MCS play a somewhat different roles across countries. In Japan, outcomes with durable LVAD support are favourable, with survival rates of 94% at 1 year and 92% at 2 years,^11^ compared with 82.8% and 79.5% at 1 and 2 years, respectively, in the MOMENTUM 3 trial.^12^ However, donor availability in Japan remains strikingly low, at only 0.9 donors per million population (pmp), compared with 44.5 pmp in the United States, 21.08 pmp in the UK, and 10.34 pmp in Germany.^13^ These differences are attributable not only to the quality of medical care, but also to geographical, cultural, and legal barriers.^14^ The heart transplant population is also markedly lower in Japan and European countries compared with the United States, with rates of only 0.93 pmp in Japan, 3.52 in the UK, 3.96 pmp in Germany, and 13.5 in the United States.^13^

The ROADMAP study highlighted that, in patients with advanced heart failure who are not dependent on inotropes, LVAD therapy can enhance functional capacity and quality of life (QOL), although it is accompanied by adverse events.^15^ Analysis of the INTERMACS registry by Suzanne et al. reported that nearly one-third of patients either died or experienced persistently poor QOL within the first year following LVAD implantation.^16^ Heart transplantation may benefit these patients by avoiding LVAD-related adverse effects and preventing deterioration in QOL.

In this study, we focused on recipients supported by durable MCS. As shown in **Table 1**, patients undergoing DCD heart transplantation experienced significantly shorter waitlist days, consistent with previous reports.^17^ Despite longer ischaemic times in DCD heart transplants, post-transplant survival and clinical outcomes were comparable to those of DBD heart transplants, even in an inverse probability-weighting cohort.

The UNOS database does not distinguish between warm and cold ischaemic times. In DCD heart procurement, a mandatory stand-off period following withdraw from life support therapy (WLST) is required for ethical reasons. This period contributes to prolonged warm ischaemic time, raising concerns about cardiac function in DCD heart.

Although outcomes in durable MCS supported patients have improved over time, complications remain common. These complications may lead to earlier heart transplantation by raising UNOS status, yet they are associated with worse outcomes. Regarding the clinical outcomes, our analysis revealed a significantly higher pretransplant infection rate in the DBD group. This disparity likely stems from the substantially longer waitlist duration observed in this cohort, which increases the risk of bridge-to-transplant complications. Conversely, the shorter waitlist time associated with accepting hearts from donors after circulatory death may offer a protective advantage by reducing time spent in a high-risk pretransplant state. Although our analysis of the UNOS dataset could not directly evaluate post-transplant haemodynamic parameters or right ventricular function, the observed difference in liver failure-related mortality is a finding that warrants further investigation. It is possible that subtle variations in early post-transplant haemodynamics or ischaemic stress—factors previously associated with hepatic complications in heart transplantation—might have played a role. However, these mechanistic explanations remain speculative within the scope of our data, and prospective studies with granular haemodynamic monitoring are required to clarify these associations.

As previously discussed, the introduction of DCD heart transplantation increases the overall number of transplants and thus provides more opportunities for patients to receive life-saving treatment, not only in the United States but internationally, including Japan and European countries. Even though durable MCS patients are now assigned lower priority under the current UNOS allocation system, our findings suggest that favourable outcomes with DCD heart transplantation in this population may justify broader implementation and help expand access to transplant for these candidates.

Our study has several limitations. First, as an analysis of the UNOS registry, this study is subject to the inherent constraints of large-scale retrospective databases, including potential missing data. Furthermore, granular data regarding DCD-specific procurement protocols and intraoperative techniques—such as the use of Normothermic regional perfusion (NRP) or Direct procurement and perfusion (DPP), myocardial protection/cardioplegia composition, and transplant duration (skin-to-skin time)—were not consistently available. Consequently, we could not evaluate the impact of these evolving technologies and perioperative variables on post-transplant outcomes; these data constraints may, therefore, limit our interpretation of ischaemic injury. Second, the DCD cohort was relatively small, resulting in a post hoc power of 0.171 for 1-year survival. This indicates the study was underpowered to detect subtle clinical differences, and the results should be interpreted with caution. Third, profound selection bias and baseline imbalances are inherent to early DCD programs, as evidenced by the 10:1 ratio between the DBD and DCD cohorts. DCD donors were significantly younger and more frequently male, and the types of durable MCS were unbalanced, with a higher prevalence of HeartMate 3 in the DCD group. Although limited data availability prevented a detailed analysis of post-transplant complications such as de novo dialysis or severe primary graft dysfunction requiring extracorporeal support, these factors are well known to influence survival as reported in recent literature.^18^^,^^19^ While providing a comprehensive overview of national outcomes, our findings highlight the critical need for more detailed donor and perioperative data to fully evaluate DCD heart transplantation.

## CONCLUSION

In conclusion, utilizing DCD donors significantly reduces waitlist duration for patients bridged with durable MCS. Although early post-transplant survival was statistically similar, drawing definitive conclusions regarding survival equivalence involves several limitations, including the small DCD cohort size, inherent selection bias, and a lack of granular donor and procedural data. While the potential for DCD transplantation to mitigate waitlist mortality in this specific high-risk subgroup is promising, a highly cautious approach with rigorous recipient and donor selection remains essential. Further larger comparative studies with comprehensive donor characteristics are strictly required to evaluate the safety profile of DCD hearts before they can be universally adopted.

## Supplementary Material

ivag214_Supplementary_Data

## Data Availability

This study utilized data from the UNOS database. Access to these data is subject to restrictions and was obtained under a data use agreement for the present study; therefore, the data are not publicly available. Data may, however, be obtained from UNOS upon reasonable request and with appropriate permissions.

## Abbreviations

BMI: body math index
CF-LVAD: continuous-flow left ventricular assist device
DBD: donation after brain death
DCD: donation after circulatory death
ECMO: extra-corporeal membrane oxygenation
IABP: intra-aortic balloon pumping
MCS: mechanical circulatory support
QOL: quality of life
UNOS: United Network for Organ Sharing
VAD: ventricular assisted device
WIT: warm ischemic time
WLST: withdraw from life support therapy
